# College Students’ Feasibility and Acceptability of a Culinary Medicine and Wellness Class and Food Security and Eating Behaviors at a Minority-Serving Institution: A Pilot Study

**DOI:** 10.3390/nu17142336

**Published:** 2025-07-17

**Authors:** Zainab Alonge, Joshua Simpkins, Claire A. Spears, Alexander Kirpich, Jessica Todd, Nida I. Shaikh

**Affiliations:** 1 Department of Nutrition, Byrdine Lewis College of Nursing and Health Professions, Georgia State University, Atlanta, GA 30303, USA; zalonge1@student.gsu.edu (Z.A.); jjohnson22@gsu.edu (J.T.); 2 Office of Institutional Research, Georgia State University, Atlanta, GA 30303, USA; jsimpkins1@gsu.edu; 3 Department of Health Policy and Behavioral Sciences, School of Public Health, Georgia State University, Atlanta, GA 30303, USA; cspears@gsu.edu; 4 Department of Population Health Sciences, School of Public Health, Georgia State University, Atlanta, GA 30303, USA; akirpich@gsu.edu

**Keywords:** culinary medicine, feasibility, acceptability, food security, mental health, academic performance, college students

## Abstract

**Objective:** This study aimed to assess the feasibility and acceptability of a Culinary Medicine and Wellness (CMW) class among undergraduate college students attending a U.S. Minority-Serving Institution (MSI), as well as their food security, mental health status, and eating behaviors. **Methods:** This pre- and post-intervention study was conducted at an MSI in a Southeastern U.S. University, where students enrolled in a 15-week, three-credit CMW class equivalent to 2.5 h per week and received instruction on cooking and preparing healthy meals on a budget. The primary outcomes were acceptability and feasibility of the CMW class. Participants’ food security status, mental health status, and fruit and vegetable intake were also assessed. Program evaluation utilized thematic analysis and descriptive statistics, and trend analyses of outcomes were performed. **Results:** Eleven participants completed both surveys. The average age was 24 years, with 73% identifying as Black/African American. All participants were female and experienced low or very low food insecurity, and most reported moderate stress levels. All participants reported they would recommend the CMW class to others, with 73% rating it as excellent. Additionally, 82% felt they had learned valuable cooking and budgeting skills. **Conclusions:** The acceptability and feasibility of a CMW class among college students at an MSI suggests a promising approach to improving cooking skills, enhancing nutrition knowledge, increasing fruit and vegetable intake, and reducing stress.

## 1. Introduction

Culinary medicine courses in colleges are gaining popularity for empowering students to prepare nutritious meals on a budget [1], yet few studies have explored their feasibility and acceptability specifically among college students [1,2,3]. College students represented about 5.6% of the U.S. population in fall 2024, totaling about 19.2 million individuals [4]. They often report a need for better food preparation skills, cooking self-efficacy, and time management [5,6,7]. Minority-Serving Institutions (MSIs) may play a crucial role in offering nutrition education opportunities to students, some of whom may also be experiencing food insecurity [8]. Nationwide, food insecurity is a growing concern, with 48.6% of undergraduate college students experiencing food insecurity in 2024 [9]. Offering culinary medicine courses at an MSI may offer a systems approach with an opportunity to improve food security among college students who often experience limited access to and affordability of healthy foods [10,11].

Most studies that have examined the feasibility and acceptability of culinary medicine programs focused on specific groups such as medical students or individuals with particular health conditions [1,2,3,12]. One study found that a culinary medicine elective for medical students at Northwestern University improved their confidence in nutrition counseling, cooking skills, and dietary behaviors, with high feasibility and acceptability demonstrated through strong attendance and retention rates [3]. Another study among 23 gastroenterology fellows at the University of California, Irvine, found a tailored culinary medicine course useful for addressing nutrition-related gastrointestinal disorders, though maintaining participant engagement throughout the program was a challenge [2]. A scoping review of 23 studies conducted in U.S. and 1 in Canada on culinary medicine in medical training highlighted improved nutritional knowledge, skills, and confidence among participants, despite variations in delivery methods and inconsistent long-term follow-up [1]. Additionally, a pilot culinary medicine program in Australia involving eight adults with mild to moderate intellectual disabilities demonstrated high acceptability, engagement, and preliminary improvements in diet quality and cooking confidence [12].

Culinary medicine blends practical cooking skills with scientific insights on how nutrition and dietary patterns impact health [13,14]. Studies have shown that participation in culinary medicine programs can improve food security, alleviate stress, and enhance culinary confidence [15]. Among college students, these classes have also been linked to increased frequency of meal preparation [16]. Culinary medicine classes on college campuses may also reduce perceived stress by equipping students with skills related to practical cooking, meal preparation, and food shopping that promote healthier eating habits, which may in turn improve mental well-being and resilience [15]. In addition to enhancing cooking skills, there have been reports of positive dietary changes, including increased fruit and vegetable intake, which are crucial for improving students’ overall health and well-being [17]. Some research suggests that improved diet quality is inversely associated with stress and depressive symptoms, though findings are mixed, with some reporting no significant association [18,19,20]. College students, who often struggle with time management and prioritize convenience and cost over nutrition, may benefit from these classes not only in terms of mental health and food security but potentially also in their academic performance.

This study aimed to assess the feasibility and acceptability of a Culinary Medicine and Wellness Class (CMW) among undergraduate college students attending an MSI in the U.S. and also to document their food security, eating behaviors, and mental health status. We hypothesized that participants would find the CMW class feasible and acceptable. Given that this CMW class was offered at an MSI, we also hypothesized that the majority of the class participants would report experiencing food insecurity.

## 2. Materials and Methods

### 2.1. Study Sample and Recruitment

This was an exploratory pilot study with a pre–post design. All undergraduate college students who were enrolled in a 15-week 3-credit Culinary Medicine and Wellness (CMW) class, which is equivalent to 2.5 h per week, at a large urban public university in the U.S. in spring 2024, were invited to participate. The large urban public university, also considered an MSI, had 27,610 undergraduate students in fall 2023 [21]. The CMW class was initially developed in 2019 and is taught every fall and spring semester. Any enrolled undergraduate student can take the CMW class. It uses a flipped classroom approach where students are assigned lectures and activities related to the Mediterranean diet and lifestyle to complete before coming to class each week. During weekly sessions in the teaching kitchen, they apply what they learned from the modules, develop knife skills, practice cooking techniques, and engage in discussions to deepen their understanding. The teaching kitchen classroom can accommodate a maximum of 19 students with one instructor. There are six small kitchens within the teaching kitchen, each with a sink, stove, smallware, and equipment. At the beginning of the semester, the class is divided into 3–4 students per kitchen, who will remain in that group for the entire semester. Each week, students prepare recipes that align with the module for the week. For example, when students learn about the macronutrient fat, they prepare recipes that contain healthy fats such as homemade salad dressings, yogurt-based dips, oven “fried” chicken tenders, and chocolate avocado pudding. The goal is to show students that healthy foods can taste great and resemble foods they like by modifying commonly used ingredients and preparation methods. Each semester, two–three sections of the class are offered. In spring 2024, all 46 undergraduate students who were enrolled in the CMW class were invited to participate in the study. This study was conducted following approval from the University’s Institutional Review Board under IRB number H22316. The university’s Office of Institutional Research (OIR) sent recruitment emails with a link to a Qualtrics baseline survey to all students enrolled in the CMW class in spring 2024.

### 2.2. Data Collection and Survey Instruments

Participants completed the pre- and post-surveys at the beginning and end of the semester, respectively. The first section of each survey included a consent form to confirm voluntary participation in the study. The pre-CMW class assessment survey (baseline survey) was sent in the second week of the spring 2024 semester, and the post-CMW class assessment survey (endline survey) was sent within one week of class completion in late April. Participants’ survey responses were linked with data on their academic performance [term and cumulative Grade Point Average (GPA)], enrollment, and socio-demographic characteristics provided by the university’s OIR. The surveys were developed using a combination of validated scale instruments from the literature and researcher-generated questions. The domains included program evaluation, food security status, mental health, eating and cooking behaviors, and fruit and vegetable intake. Mental health measures included stress, anxiety, depression, coping, and resilience.

Food security status was measured using the United States Department of Agriculture (USDA) 10-item Food Security Module [22]. The 10-item Perceived Stress Scale (PSS) was used to measure the perceived stress of the participants [23]. The severity of anxiety was measured with the Generalized Anxiety Disorder tool (GAD-7) [24]. Resilience was assessed with the Connor–Davidson Resilience Scale (CD-RISC), a 25-item tool in which higher summed scores reflect higher resilience [25]. Coping strategies were assessed using the Brief COPE Inventory; 28 items were categorized into three main categories including problem-focused (i.e., active coping, use of instrumental support, positive reframing, planning; these tend to be more adaptive coping strategies), emotion-focused (i.e., use of emotional support, venting, acceptance, self-blame), and avoidant coping (i.e., self-distraction, denial, substance use, behavioral disengagement; these tend to be more maladaptive coping strategies) [26,27]. Whereas problem-focused coping strategies tend to be more adaptive and avoidant strategies tend to be maladaptive, the emotion-focused coping subscale is not uniformly associated with better or worse psychosocial functioning. Depression risk was assessed using the Center for Epidemiologic Studies Depression (CES-D) Scale, a 20-item measure [28].

A program evaluation was conducted at the endline of the study using researcher-generated questions to gather feedback from the participants on the feasibility and acceptability of the CMW class. The feasibility and acceptability were assessed through multiple indicators including overall satisfaction with the class, perceived quality of instruction, skill development (knife skills), and confidence in applying the skills learned.

### 2.3. Data Analysis

De-identified survey data and institutional data were processed and analyzed using SAS (version 9.4). Descriptive analyses were conducted to summarize baseline participant characteristics as well as quantitative scores on baseline and endline mental health measures (e.g., CES-D, GAD-7, PSS), food security status, fruit and vegetable intake, academic performance (GPA), and program evaluations. Descriptive statistics were computed. For the analysis, Fisher’s exact tests were used for categorical variables, and paired *t*-tests and Wilcoxon rank-sum tests (as alternatives) were used for continuous variables. The corresponding *p*-values were reported. Chi-squared tests for categorical variables were not performed due to the limited sample size and the resulting unreliability of chi-squared approximations. Results from the Wilcoxon rank-sum tests, included for comparison, are available in the Supplementary Materials. Qualitative data from open-ended program evaluation questions were analyzed in Excel. These responses were organized, coded, and examined for recurring themes and patterns [29].

## 3. Results

Of the 46 students invited to participate, 25 completed either the baseline or endline survey, and 11 of those completed both surveys. The summary means were reported together with the standard deviations in parentheses. Table 1 shows the baseline demographic characteristics of the 11 participants who completed both the baseline and endline surveys. Overall, the average age was 24.1 (7.3), all participants were female, 72.7% identified as Black or African American, 27.3% were first-generation participants, 72.7% lived off-campus, 27.3% reported being single, 54.5% were Pell Grant-eligible, and none were enrolled in the Supplemental Nutrition Assistance Program (SNAP). At baseline, the mean GPA was 3.16 (0.7), with 45.5% participants’ GPA in the 3.00–3.99 range. At endline, the mean GPA was 3.30 (0.6), with 63.6% of participants in the 3.00–3.99 GPA range. Most participants received an A or A+ in their culinary medicine class (81.8%).

Table 2 shows participants’ endline ratings of the CMW class. All participants reported liking the class “a lot” and would “definitely recommend” the class to others. The majority (72.7%) rated the class quality as “excellent”, with all others rating it “very good”. Most of the participants (81.9%) either agreed or completely agreed that they learned how to cook and also learned how to cook on a budget. About 45.5% rated their knife skills as “excellent”, while 54.5% rated them as “very good”. Confidence in practicing the learned skills varied: 54.5% reported that they were “extremely confident”, 18.2% were “very confident”, and 27.3% reported being “moderately confident”.

Based on the open-ended program evaluation questions, participants had a positive experience in the CMW class; responses reflecting overall contentment included broad statements of liking “Everything” and positive comments about the instructors, teaching assistants, and classmates in addition to the knowledge and skills learned. While the sample size was small, the majority of participants did not indicate any negatives about the course. In particular, when asked about their recommendations to improve the class, most responded “none” or “N/A”.

Four main themes emerged from the qualitative responses. First, in theme one, many participants appreciated the opportunity to engage directly in cooking. They valued the hands-on experience of trying out new recipes and techniques. They highlighted their enjoyment of “hands-on cooking”; for example, one participant said what they liked best about the class was “the opportunity we were given to cook and grow in the classroom”. Second, participants appreciated the variety and nature of the recipes taught. Participants enjoyed learning new and diverse recipes, particularly those that were not typically “American”. The feedback emphasized the value of exploring “different and unique types of foods” and appreciating the “variety of recipes”. One participant explained, “I really liked being able to discover different and unique types of foods and being able to expand my palate”. The class had a notable impact on participants’ perceived cooking skills and their ability to make healthy food choices. Third, participant feedback highlighted their appreciation of learning healthy cooking habits that could be incorporated into everyday life. For example, one participant said, “I liked learning how to healthify basic recipes”. Another explained, “I’ve learned to really examine what I eat and to make small changes daily to incorporate more nutritious choices, i.e., food swaps”. Many participants reported increased confidence in cooking, a greater understanding of nutrition, and a willingness to try new foods. Comments included “Yes, I make more meals at home now” and “This class benefited me because I learned new nutrition facts and tried new foods”. One participant noted that the class fostered a positive atmosphere that contributed to a sense of peace and happiness. Another participant noted improvement in her ability to speak in public, reporting that “I am a very quiet person, but this class was a great space where I felt comfortable speaking”. Fourth, participants provided suggestions to improve the class, which included expanding the variety of meals.

The few criticisms mentioned were related to the quality and practicality of some of the food items prepared, such as dissatisfaction with some of the foods prepared and concerns over food swaps. One participant stated “Some of the health swaps were not completely realistic like Greek yogurt as a ranch swap”. Another participant recommended “a wider variety of meals”, while another asked for “more breakfast recommendations”. Another suggestion was to “increase the cost per serving on the final project in relation to the rising cost of groceries; USD 3 per serving was limiting”. The final project required participants to find a healthy recipe and prepare a dish, with the cost limited to no more than USD 3 per serving. Besides adjusting the budget for the final project dish, participants suggested “more time for cooking”.

Table 3 shows the quantitative results on pre–post food security and mental health (n = 11). At baseline, 81.8% of the participants reported low food security (score 3–5), and 18.2% reported very low food security (score 6–10), with a mean score of 5.72 (1.7). At endline, 72.7% of the participants reported low food security, and 27.3% reported very low food security, with a mean score of 5.45 (0.9). Low stress (score 0–13) was reported by about 27% of participants at baseline and 9.1% at endline. Participants reported 55% moderate stress (score 14–26) at baseline and 72.7% at endline. The mean perceived stress score was 19.27 at baseline and 17.54 at endline, indicating moderate stress at both time points. There was no change in the distribution of resilience scores at baseline and endline; 72.7% reported high resilience (score > 25.5) both at baseline and endline. At baseline, the participants had a mean anxiety score of 7.73 (5.4), and 7.00 (5.4) at endline, indicating mild anxiety (score 5–9) at both time points. The proportion of participants experiencing minimal anxiety (score 0–4) trended up from 27.3% to 36.4%, while the proportion of participants experiencing moderate anxiety (score 10–14) showed a downward trend from 18.2% to 9.1%. For depression risk, 27.3% of participants were at risk for depression (score > 16) at baseline, with a mean of 17.67 (14.9). At baseline, a lower proportion of participants (18.2%) were at risk for depression, with a mean score of 17.00 (14.3). Participants relied more on problem-focused and emotion-focused coping strategies, with minimal change across both time points.

Table 4 shows the results regarding fruit and vegetable consumption (n = 11). At baseline, 54.5% of participants consumed 1–2 servings of any type of fruit per day, while 27.3% reported consuming 3–4 servings. By endline, participants consuming 1–2 servings of any type of fruit per day trended up from 27.3% to 63.6%, while there was a downward trend in the number of participants consuming 3–4 servings of all types of fruits per day (from 27.3% at baseline to 18.2% at endline). There was an upward trend in the proportion of participants consuming fresh fruits 3–4 times per week from 27.3% to 45.5%. In terms of vegetable intake, we found an upward trend in the proportion of participants who consumed 3–4 servings of any type of vegetable per day, increasing from 36.4% at baseline to 72.7% at endline, as well as in the proportion of participants who consumed fresh vegetables 1–2 times per week, increasing from 18.2% at baseline to 45.5% at endline. On the other hand, the proportion of participants that consumed fresh vegetables daily trended downward from 27.3% to 9.1%, and the frequency of canned vegetable consumption showed a slight downward trend at endline compared to baseline, while there appeared to be no change in consumption of frozen vegetables during the study period.

Table 5 shows the pre–post eating, cooking, and food purchasing behaviors of participants (n = 11). Fisher’s exact tests were performed for categorical variables and paired *t*-tests for continuous variables. Most meals were eaten at home, trending upward from 72.7% at baseline to 90.9% at endline. Participants reportedly ate an average of 2.2 (0.8) meals per day at baseline and 2.1 (0.6) meals per day at endline. By endline, the percentage of participants consuming two meals had trended upwards to 63.6%, with those consuming three meals trending downwards to 27.3%. Participants took an average of 1.9 (0.9) snacks per day at baseline and 1.82 (0.8) snacks per day at endline. The most commonly skipped meal by the participants was breakfast at both baseline (n = 5, 45.5%) and endline (n = 6, 54.5%). Average daily meal-skipping frequency trended downwards from 45.5% at baseline to 36.4% at endline. There was a shift in the type of cooking method commonly used: the proportion of participants that used stove-top cooking experienced an upward trend from 81.9% at baseline to 100.0% at endline. There was an upward trend in the proportion of participants using baking as a means of food preparation, from 36.4% at baseline to 72.7% at endline. Regarding food purchasing behaviors, all participants purchased food items from grocery stores. Other response options, such as shopping at convenience stores, not typically shopping, and “Other (please specify)”, were not selected. Food pantries were barely used: the proportion of participants who had never visited a food pantry trended upwards from 63.4% at baseline to 81.8% at endline.

## 4. Discussion

Overall, undergraduate students attending a culinary medicine class at a large, public, Minority-Serving Institution in the U.S. provided positive feedback reporting that the class taught them several skills such as learning not only how to cook and knife skills but also cooking on a budget. Participants reported they would practice what they had learned in the class. A high level of satisfaction was expressed by participants both for the class and for the skills learned. They expressed they were better equipped to prepare healthy recipes, consistent with the findings of a study involving 84 health professional students taking an 8-week culinary medicine class at the University of Utah, where they reported significant improvements in their ability to prepare healthy meals [30]. Many participants in our study also highlighted that they had learned to cook on a budget, which is crucial since financial and time constraints are known barriers to healthy eating among students and also lead to less frequent cooking among students experiencing food insecurity [7]. The strong confidence expressed in applying their new cooking skills further puts into perspective how the class has the ability to empower students to choose sustainable cooking practices.

In addition to examining the feasibility and acceptability of the culinary medicine class among college students, we evaluated their food security, mental health, academic performance, fruit and vegetable intake, and eating behaviors. All 11 participants were experiencing either low or very low food security. Among a convenience sample of 400 students at the same university where the study was conducted, 59% were experiencing food insecurity [31]. However, even though all participants in our pilot study were experiencing food insecurity, only 55% were eligible to receive a Federal Pell Grant, and none reported being enrolled in SNAP or using food pantries and food giveaways. The SNAP benefits are designed to help low-income households pay for food, but students must meet additional criteria to qualify, such as working at least 20 h a week at a paid job [32]. While beyond the scope of this study, institutions could focus on understanding college students’ awareness and barriers to utilization of SNAP as well as other food resources and assist those who meet the specific eligibility requirements in signing up for these programs.

Food insecurity has been found to be negatively associated with GPA among college students. Our study showed an upward trend in overall GPA at the end of the CMW; however, we did not conduct statistical tests. The sample size was small, the control group was not available, and the classes the participants took during the studied period may have varied in complexity, influencing potential changes in GPA due to other reasons which may have introduced some ambiguity. While these findings are not conclusive, feedback from participants suggests that this class may have indirectly helped maintain their academic performance by possibly contributing to reduced stress levels and providing tangible learning skills related to cooking and budgeting. It is important to note the diversity of the class: participants took this class at different points in their undergraduate journey and represented different majors including nutrition, computer science, nursing, psychology, and film and media, to name a few which might have affected their experiences and academic outcomes. Although no previous research has investigated the impact of a culinary medicine class on GPA, this exploratory study fills an important gap by providing new information about the academic performance of a diverse undergraduate population, who were experiencing low or very low food security.

A majority of participants showed high resilience and low risk for depressive symptoms at baseline and endline. In terms of coping strategies, the participants tended to use more problem-focused strategies (e.g., active coping, planning) and fewer avoidant strategies (e.g., denial, substance use), which indicates a favorable pattern of adaptive coping and aligns with scores reported in other non-clinical samples [27,33]. A study of 65 undergraduate students enrolled in a four-session cooking curriculum at two land grant institutions found an improvement in the number of days [13.7 (9.8) to 11.7 (9.5) days] the participants were worried, tense, or anxious using CDC’s Health Days Module [34,35,36,37,38]. Although our study used a different duration and different validated questionnaires to assess anxiety, we found a downward trend in mean GAD score from 7.7 (5.4) at baseline to 7.0 (5.4) at endline, which is not necessarily a clinically meaningful change as both fall within the “mild anxiety” range [24]. Students’ level of stress may relate to the struggle of juggling academic demands with financial food security and other challenges as documented in other studies as well [39,40]. A study of 1138 freshman students at a public metropolitan university found that students experiencing financial difficulties faced an increased level of stress using a four-question perceived stress questionnaire [41]. The mean stress score in our study experienced a downtrend from 19.3 (7.7) to 17.5 (7.5), suggesting that students experienced moderate levels of stress both before and after the class. This finding aligns with slight reductions in perceived stress in a study among 171 undergraduates in a large public university in California who completed a culinary medicine class from 19.7 (5.9) pre-intervention to 18.1 (6.0) post-intervention [15]. Larger studies are needed to determine any significant changes in stress, but it is possible that a culinary medicine class could reduce students’ perceived stress by promoting empowerment through increased practical cooking and nutrition knowledge, enabling students to make better dietary choices and foster a greater sense of control of their health. More research with larger samples is needed to examine changes over time and mediators and moderators of those changes.

We observed small changes in the total vegetable servings eaten per day and the upward trend in frozen vegetable consumption per week among students after attending the CMW class. While there was no change in the trend of total fruit servings per day, fresh fruit and canned fruit consumption per week trended upwards as participants were now consuming them more times per week. The previous literature has also shown that food literacy and culinary skills interventions were positively associated with increased intake of nutrient-dense foods such as fruits, vegetables, and whole grains among college students [16,17]. These results suggest the culinary medicine class may have equipped participants with practical cooking skills and food literacy, enabling them to prepare and consume more nutrient-dense foods like vegetables and fruits, even within the time and access constraints typical of a college environment.

This study was not without limitations. First, this was a pilot pre–post exploratory study without a control group; as such, we were not able to evaluate the effects of the intervention on study outcomes. More specifically, the presented study was initially planned as a preliminary pilot study with a relatively small sample size and without a control group, which inherently limited the strength of its conclusions. As a result, the findings from this study (as well as from any pilot study without a control group) should be interpreted with caution, and relevant limitations should be well understood. In the meantime, the presented pilot study results were very promising, since program acceptability was high, and quantitative results suggest the intervention promotes the development of critical culinary skills. Next, the response rate was low. Out of the 46 students enrolled in the CMW class in spring 2024, 25 students (54.3%) participated in either of the two surveys, but only 11 (23.9%) students completed both surveys. As this was an exploratory pilot study, we did not have funding to offer incentives. Participants were given extra credit for study participation. To improve response rates, future efforts could include collecting data over multiple semesters to have a larger sample size and offering incentives for participating in the study. This pilot study allowed for only one semester or 15 weeks of data collection, but one semester may have been a short period to capture changes in some of the outcomes including food security, mental health, and fruit and vegetable intake. A second limitation was that the small sample size of 11 participants allowed us to examine only the trends in the study outcomes. Even so, this pilot study is the first to provide insights into the feasibility and acceptability of a culinary medicine program at an MSI, and the assessments of multiple interrelated outcomes related to food security, mental health, and dietary habits suggest potential benefits of the course. Another limitation is the potential for response bias, particularly in the domains of mental health, fruit and vegetable intake, and eating behaviors, as participants may have underreported or overreported their experiences due to social desirability or other factors. Also, our pilot study was limited to undergraduate students. Graduate students were excluded from this study as the graduate portion of the program is open only to graduate nutrition students and has a different focus. Future work could evaluate the program’s effectiveness and applicability for graduate students. They could track participants 6–12 months post-intervention to assess whether significant changes in cooking confidence and dietary habits are sustained over time. Also, future work could measure long-term changes in students’ food security, stress levels, and academic performance.

Despite the limitations, this study had several strengths. We assessed the feasibility and acceptability of an established CMW class for undergraduate college students at a large public university also recognized as an MSI. This is particularly important because MSIs serve large proportions of students from racial and ethnic minoritized groups who are at increased risk of food insecurity, poor dietary quality, and health disparities [10,11]. We made use of an existing class that is available campus-wide each semester to all enrolled undergraduate students without requiring students to take any pre-requisites. The positive feedback from the participants about the CMW class’s ability to teach critical culinary skills and nutrition education, as well as the high course quality ratings, could be used as drivers to scale this class up. To offer the benefits of completing a culinary medicine class to more students each semester, other institutions (especially MSIs), could consider investing in a larger teaching kitchen space and offering more sections of the class. Another strength was our use of validated measures of food security, anxiety, depressive symptoms, and coping strategies for the college student population.

## 5. Conclusions

Findings from this pilot study support further research into the feasibility and acceptability of a culinary medicine class among undergraduate college students at a public MSI. Students provided strong ratings in terms of the quality of the course and appreciated improving their cooking skills and food budgeting practices. Higher education institutions might consider the development of new or the expansion of existing culinary medicine classes on college campuses to interactively teach students nutrition education and other valuable skills, such as not only to cook but also to cook on a budget, in a teaching laboratory. This program could be scaled up at other MSIs and colleges to reach a wider and more diverse student population to improve food security and equip students with practical tools to enhance their food choices and dietary habits. Tangible skills learned in culinary medicine classes could help students cope with and better manage their stress, while at the same time enhancing their capability to manage meal preparation and identify healthier food options. These skills would be especially valuable for low-income students experiencing food insecurity. Future studies should focus on the long-term impact of the culinary medicine class on the outcomes measured in the current study, consider including a control group, and expand the eligibility criteria to include graduate students.

## Figures and Tables

**Table 1:** Socio-demographic characteristics and academic performance of college students attending a culinary medicine class at a public university in Southern U.S. (n = 11).

Characteristics	N (%)
Age (years)	
Mean (SD)	24.1 (7.3)
Gender	
Female	11 (100.0)
Race	
Black or African American	8 (72.7)
Asian	3 (27.3)
Ethnicity	
Not Hispanic or Latino	11 (100.0)
First generation	
Yes	3 (27.3)
No	8 (72.7)
Father’s education Level	
High School	5 (45.5)
College Degree	4 (36.4)
Not Reported	2 (18.2)
Mother’s education Level	
High School	4 (36.4)
College Degree	5 (45.5)
Not Reported	2 (18.2)
Marital status	
Single	3 (27.3)
Not Reported	8 (72.7)
Pell Grant-eligible	
Yes	6 (54.5)
No	5 (45.5)
Housing	
On Campus	3 (27.3)
Off Campus	8 (72.7)
Baseline GPA (all courses)	
2–2.99	4 (36.4)
3–3.99	5 (45.5)
>4	1 (9.1)
Missing	1 (9.1)
Mean (SD)	3.16 (0.7)
Endline GPA (all courses)	
2–2.99	3 (27.3)
3–3.99	7 (63.6)
>4	1 (9.1)
Mean (SD)	3.30 (0.6)
Culinary Medicine Class grade	
A+	8 (72.7)
A	1 (9.1)
B+	1 (9.1)
B	1 (9.1)
Total credit hours taken	
<20	1 (9.1)
20–50	0
50–100	4 (36.4)
100–120	6 (54.5)
Mean (SD)	89.90 (29.7)

**Table 2:** Acceptability and feasibility of a culinary medicine class among college students at a public university in the Southern U.S. (n = 11).

Characteristics	Endline N (%)
Liked class a lot	11 (100.0)
Class quality	
Excellent	8 (72.7)
Very Good	3 (27.3)
Learned how to cook	
Completely Agree	5 (45.5)
Agree	4 (36.4)
Disagree	2 (18.2)
Learned to cook on a budget	
Completely Agree	5 (45.5)
Agree	4 (36.4)
Neutral	1 (9.1)
Disagree	1 (9.1)
Knife skills rating	
Excellent	5 (45.5)
Very Good	6 (54.5)
Will recommend class	
Would Definitely	11 (100.0)
Will practice skills learned	
Extremely Confident	6 (54.5)
Very Confident	2 (18.2)
Moderately Confident	3 (27.3)

**Table 3:** Food security status and mental health indicators of college students attending a culinary medicine class at a public university in Southern U.S (n = 11).

Outcomes	Baseline N (%)	Endline N (%)	*p*-Value
Food security			
Low food security (score 3–5)	9 (81.8)	8 (72.7)	
Very low food security (score 6–10)	2 (18.2)	3 (27.3)	
Mean (SD)	5.72 (1.7)	5.45 (0.9)	‡ 0.34
Perceived stress scale			
Low stress (score 0–13)	1 (9.1)	3 (27.3)	
Moderate stress (score 14–26)	8 (72.7)	6 (54.5)	
High stress (score 27–40)	2 (18.2)	2 (18.2)	
Mean (SD)	19.27 (7.7)	17.54 (7.5)	‡ 0.26
Generalized anxiety disorder scale			
Minimal anxiety (score 0–4)	3 (27.3)	4 (36.4)	
Mild anxiety (score 5–9)	5 (45.5)	5 (45.5)	
Moderate anxiety score (10–14)	2 (18.2)	1 (9.1)	
Severe anxiety (score ≥ 15)	1 (9.1)	1 (9.1)	
Mean (SD)	7.73 (5.4)	7.00 (5.4)	‡ 0.55
Connor–Davidson resilience scale			
High resilience (score > 25.5)	8 (72.7)	8 (72.7)	
Low resilience (score ≤ 25.5)	3 (27.3)	3 (27.3)	
Mean (SD)	32.72 (9.7)	33.30 (9.9)	‡ 0.22
Center for epidemiologic studies depression scale			
Low depression risk (score < 16)	8 (72.7)	9 (81.8)	
At risk for depression (score ≥ 16)	3 (27.3)	2 (18.2)	
Mean (SD)	17.67 (14.9)	17.00 (14.3)	‡ 0.42
Coping orientation to problems experienced inventory [mean (SD)]			
Problem-focused coping (score 1–4)	2.58 (0.8)	2.54 (0.7)	‡ 0.51
Emotion-focused coping (score 1–4)	2.23 (0.7)	2.31 (0.7)	‡ 0.79
Avoidant coping (score 1–4)	1.75 (0.6)	1.74 (0.7)	‡ 0.82

‡ *p*-value for paired *t*-test.

**Table 4:** Fruit and vegetable consumption among college students attending a culinary medicine class at a public university in Southern U.S. (n = 11).

Outcomes	Baseline N (%)	Endline N (%)	*p*-Value
Total fruit servings (serving/day)			
3–4 servings	3 (27.3)	2 (18.2)	
1–2 servings	6 (54.5)	7 (63.6)	
None	0 (0)	2 (18.2)	
Missing	2 (18.2)	0 (0)	† 0.98
Fresh fruit consumption (times/week)			
Daily	2 (18.2)	1 (9.1)	
3–4 times a week	3 (27.3)	5 (45.5)	
1–2 times a week	5 (45.5)	4 (36.4)	
Never	0 (0)	1 (9.1)	
Missing	1 (9.1)	0 (0)	
Mean (SD)	3.2 (2.2)	2.77 (1.9)	‡ 0.73
Canned fruit consumption (times/week)			
Daily	0 (0)	0 (0)	
3–4 times a week	0 (0)	0 (0)	
1–2 times a week	5 (45.5)	7 (64)	
Never	5 (45.5)	4 (36)	
Missing	1 (9.1)	0 (0)	
Mean (SD)	0.75 (0.8)	0.95 (0.8)	‡ 0.59
Frozen fruit consumption (times/week)			
Daily	0 (0)	0 (0)	
3–4 times a week	0 (0)	0 (0)	
1–2 times a week	4 (36.4)	5 (45.5)	
Never	5 (45.5)	6 (54.5)	
Missing	2 (18.2)	0 (0)	
Mean (SD)	0.67 (0.8)	0.68 (0.8)	‡ 0.26
Types of fruits consumed			
Apples	9 (81.8)	8 (72.7)	
Bananas	6 (54.5)	5 (45.5)	
Berries	8 (72.7)	10 (90.9)	
Citrus fruits (oranges, lemons, etc.)	6 (54.5)	8 (72.7)	
Other (grape, mango, pineapple)	1 (9.1)	2 (18)	
Total vegetable servings (servings/day)			
3–4 servings	4 (36.4)	8 (72.7)	
1–2 servings	5 (45.5)	3 (27.3)	
None	0 (0)	0 (0)	
Missing	2 (18.2)	0 (0)	†* 0.03
Fresh vegetable consumption (times/week)			
Daily	3 (27.3)	1 (9.1)	
3–4 times a week	4 (36.4)	4 (36.4)	
1–2 times a week	3 (27.3)	5 (45.5)	
Never	0 (0)	1 (9.1)	
Missing	1 (9.1)	0 (0)	
Mean (SD)	3.95 (2.2)	2.59 (1.9)	‡ 0.05
Canned vegetable consumption (times/week)			
Daily	0 (0)	0	
3–4 times a week	3 (27.3)	2 (18.2)	
1–2 times a week	3 (27.3)	5 (45.5)	
Never	3 (27.3)	4 (36.4)	
Missing	2 (18.2)	0 (0)	
Mean (SD)	1.67 (1.5)	1.30 (1.3)	‡ 0.35
Frozen vegetable consumption (times/week)			
Daily	1 (9.1)	1 (9.1)	
3–4 times a week	4 (36.4)	2 (18.2)	
1–2 times a week	2 (18.2)	6 (54.5)	
Never	3 (27.3)	2 (18.2)	
Missing	1 (9.1)	0 (0)	
Mean (SD)	4.15 (4.7)	4.72 (5.1)	‡ 0.47
Types of vegetables consumed			
Leafy greens (spinach, kale, lettuce)	9 (81.8)	9 (81.8)	
Cruciferous vegetables (broccoli, cauliflower)	9 (81.8)	9 (81.8)	
Root vegetables (carrots, potatoes)	9 (81.8)	9 (81.8)	
Bell peppers	4 (36.4)	9 (81.8)	
Other (brussels, asparagus)	1 (9.1)	0 (0)	

* Significant at *p* < 0.05. ‡ *p*-value for paired *t*-test; † *p*-value for Fisher’s exact test; no tests where participants could select more than one option.

**Table 5:** Eating, cooking, and food purchase behaviors of college students attending a culinary medicine class at a public university in Southern U.S (n = 11).

Characteristics	Baseline N (%)	Endline N (%)	*p*-Value
Number of meals per day			
1	2 (18.2)	1 (9.1)	
2	4 (36.4)	7 (63.6)	
3	4 (36.4)	3 (27.3)	
≥4	0 (0)	0 (0)	
Missing	1 (9.1)	0 (0)	‡ 0.91
Mean (SD)	2.2 (0.8)	2.18 (0.6)	
Number of snacks per day			
1	4 (46.4)	5 (45.5)	
2	4 (36.4)	3 (27.3)	
3	1 (9.1)	3 (27.3)	
≥4	1 (9.1)	0 (0)	
Missing	1 (9.1)	0 (0)	
Mean (SD)	1.9 (0.9)	1.82 (0.8)	‡ 0.34
Meals skipped frequency			
Daily	5 (45.5)	4 (36.4)	
Weekly	2 (27.3)	2 (18.2)	
Bi-weekly	1 (9.6)	3 (27.3)	
Rarely	2 (18.2)	2 (18.2)	
Missing	1 (9.1)	0 (0.0)	† 0.10
Meal skipped most often			
Breakfast	5 (45.5)	6 (54.5)	
Lunch	4 (36.4)	5 (45.5)	
Dinner	1 (9.1)	0 (0)	
Missing	1 (9.1)	0 (0)	† 0.06
Where meals are eaten (pick all that apply)			
Home	8 (72.7)	10 (90.9)	
Off-campus restaurants	1 (9.1)	2 (18.2)	
Dining hall	1 (9.1)	1 (9.1)	
On-campus retail locations	1 (9.1)	1 (9.1)	
Cooking method (pick all that apply)			
Stove-top cooking	9 (81.9)	11(100.0)	
Micro-wave cooking	6 (54.5)	4 (36.4)	
Micro-wave (heat and eat)	5 (45.5)	6 (54.5)	
Bake	4 (36.4)	8 (72.7)	
No cooking required (e.g., toss salads, sandwiches)	4 (36.4)	6 (54.5)	
Air fryer	1 (9.1)	1 (9.1)	
Receive SNAP			
Yes	0 (0)	0 (0)	
No	10 (90.9)	11 (100)	
Missing	1 (9.1)	0 (0)	
Location where food is purchased			
Grocery Store	10 (90.9)	11 (100.0)	
Missing	1 (9.1)	0 (0)	
Frequency of receiving food from a food giveaway			
Rarely	1 (9.1)	1 (9.1)	
Never	9 (81.8)	10 (90.9)	
Missing	1 (9.1)	0 (0)	† 0.10
Food pantry visit frequency			
Bi-weekly	1 (9.1)	1 (9.1)	
Monthly	0 (0)	1 (9.1)	
Rarely	2 (18.2)	0 (0)	
Never	7 (63.6)	9 (81.8)	
Missing	2 (18.2)	0 (0)	† 0.07
Received formal training in knife skills or culinary arts			
Yes	3 (27.3)	8 (72.7)	
No	6 (54.5)	2 (18.2)	
Missing	1 (9.1)	1 (9.1)	† 0.58
Use of knife confidence rating			
High confidence	2 (18.2)	4 (36.4)	
Very confident	5 (45.5)	5 (45.5)	
Moderate confidence	3 (27.3)	2 (18.2)	
Missing	1 (9.1)	0 (0)	† 0.60

‡ *p*-value for paired *t*-test; † *p*-value where participants could select more than one option.

## Data Availability

The raw data supporting the conclusions of this article will be made available by the authors on request.

## Supplementary Materials

The following supporting information can be downloaded at https://www.mdpi.com/article/10.3390/nu17142336/s1, Table S1: Socio-demographic Characteristics and Academic Performance of College Students Attending a Culinary Medicine Class at a Public University in Southern U.S.; Table S2: Acceptability and Feasibility of a Culinary Medicine Class among College Students at a Public University in the Southern U.S.; Table S3: Food Security Status and Mental Health Indicators of College Students Attending a Culinary Medicine Class at a Public University in Southern U.S. Table S4: Fruit and Vegetable Consumption among College Students Attending a Culinary Medicine Class at a Public University in Southern U.S.; Table S5: Eating, Cooking and Food Purchase Behaviors of College Students Attending a Culinary Medicine Class at a Public University in Southern U.S. Table S6: Food Security Status and Mental Health Indicators of College Students Attending a Culinary Medicine Class at a Public University in Southern U.S.; Table S7: Fruit and Vegetable Consumption among College Students Attending a Culinary Medicine Class at a Public University in Southern U.S.; Table S8: Eating, Cooking and Food Purchase Behaviors of College Students Attending a Culinary Medicine Class at a Public University in Southern U.S.; Table S9: Exploratory Spearman Correlations Between Endline Food Security and Selected Outcomes.
