# Supplementary material for: College Students’ Feasibility and Acceptability of a Culinary Medicine and Wellness Class and Food Security and Eating Behaviors at a Minority-Serving Institution: A Pilot Study
Source: Nutrients. 2025 Jul 17;17(14):2336. doi: 10.3390/nu17142336 (PMC12297996; doi:10.3390/nu17142336)
Supplement: Supplementary file 1 [file nutrients-17-02336-s001.zip › nutrients-3677645-supplementary-done.pdf]

nutrients

MDPI

Supplementary Tables

Table S1 - Socio-demographic Characteristics and Academic Performance of College Students Attending a Culinary Medicine Class at a Public University in Southern U.S. [n=25; N=21 (baseline) and N=15 (endline)]

Characteristics	N (%)
Age (years)	
Mean (SD)	23.2 (6.1)
Gender	
Male	4 (16.0)
Female	21 (84.0)
Race	
Black or African American	18 (72.0)
Asian	5 (20.0)
Hispanic	2 (8.0)
Ethnicity	
Not Hispanic or Latino	2 (8.0)
Hispanic or Latino	23 (88.0)
Not Reported	1 (4.0)
First generation	
Yes	5 (20.0)
No	20 (80.0)
Father's education level	
High School	8 (32.0)
College Degree	13 (52.0)
Middle School	1 (4.0)
Not Reported	3 (12.0)
Mother's education level	
High School	9 (36.0)
College Degree	9 (36.0)
Not Reported	7 (28.0)
Marital status	
Single	9 (36.0)
Not reported	16 (64.0)
Pell grant eligible	
Yes	13 (52.0)
No	12 (48.0)
Housing	
On Campus	13 (52.0)
Off Campus	12 (48.0)
Baseline GPA (all courses)	

1-1.99	2 (8.0)
2-2.99	8 (32.0)
3-3.99	11 (44.0)
>4	2 (8.0)
Missing	2 (8.0)
Mean (SD)	3.04 (0.7)
Endline GPA (all courses)	
1-1.99	1 (4.0)
2-2.99	8 (32.0)
3-3.99	13 (52.0)
>4	3 (12.0)
Mean (SD)	3.6 (1.0)
Culinary Medicine Class grade	
A	5 (20.0)
A+	11 (44.0)
B	3 (12.0)
B+	3 (12.0)
B-	1 (4.0)
C-	1 (4.0)
WF	1 (4.0)
Total credit hours taken	
<20	1 (4.0)
20-50	1 (4.0)
50-100	14 (56.0)
100 – 120	8 (32.0)
>120	1 (4.0)
Mean (SD)	86 (25.9)

Table S2 - Acceptability and Feasibility of a Culinary Medicine Class among College Students at a Public University in the Southern U.S. (n=15)

Characteristics	Endline N (%)
Liked class a lot	15 (100.0)
Class quality	
Excellent	10 (66.7)
Very Good	5 (33.3)
Learned how to cook	
Completely Agree	8 (53.3)
Agree	5 (33.3)
Disagree	2 (13.3)
Learned to cook on a budget	
Completely Agree	8 (53.3)
Agree	4 (26.7)
Neutral	2 (13.3)
Disagree	1 (6.7)
Knife skill rating	
Excellent	8 (53.3)
Very Good	7 (46.7)
Will recommend class	
Would Definitely	15 (100.0)
Will practice skills learned	
Extremely Confident	8 (53.3)
Very Confident	2 (13.3)
Moderately Confident	5 (33.3)

Table S3 - Food Security Status and Mental Health Indicators of College Students Attending a Culinary Medicine Class at a Public University in Southern U.S [n=25; N=21 (baseline) and N=15 (endline)]

Outcomes	Baseline N (%)	Endline N (%)
Food security		
Low food security (score 3-5)	14 (66.7)	10 (66.7)
Very low food security (score 6-10)	7 (33.3)	5 (33.3)
Mean (SD)	6.42 (1.5)	6.07 (0.82)
Perceived stress scale		
Low stress (score 0–13)	3 (14.3)	3 (20.0)
Moderate stress (score 14–26)	16 (76.2)	9 (60.0)
High stress (score 27–40)	2 (9.5)	3 (20.0)
Mean (SD)	18.62 (4.0)	18.80 (3.8)
Generalized anxiety disorder scale		
Minimal Anxiety (score 0-4)	5 (23.8)	7 (46.7)
Mild Anxiety (score 5-9)	9 (42.8)	5 (33.0)
Moderate Anxiety (score10-14)	5 (23.8)	2 (13.3)
Severe Anxiety (score >15)	1 (4.8)	1 (6.7)
Mean (SD)	7.53 (4.9)	6.60 (5.0)
Connor-Davidson resilience scale		
High resilience (score >25.5)	14 (66.7)	11 (73.3)
Low resilience (score < 25.5)	7 (33.3)	4 (26.7)
Mean (SD)	32.05 (9.0)	33.7 (8.2)
Center for epidemiologic studies depression scale		
Low Depression risk (score <16)	17 (81.0)	13 (86.7)
At Risk for Depression (score >16)	4 (19.0)	2 (13.3)
Mean (SD)	19.03 (13.6)	18.82 (11.59)
Coping orientation to problems experienced inventory [(Mean (SD)]		
Problem Focused Coping 1-4	2.54 (0.8)	2.52 (0.7)
Emotion Focused Coping 1-4	2.19 (0.6)	2.30 (0.5)
Avoidant Coping 1-4	1.73 (0.6)	1.76 (0.4)
Academic performance (GPA)		
Mean (SD)	3.04 (0.7)	3.41 (0.7)

Table S4 - Fruit and Vegetable Consumption among College Students Attending a Culinary Medicine Class at a Public University in Southern U.S [n=25; N=21 (baseline) and N=15 (endline)]

Outcomes	Baseline N (%)	Endline N (%)
Total fruit servings (serving/day)		
5 or more servings	1 (4.8)	0 (0)
3 - 4 servings	3 (14.3)	9 (60.0)
1 – 2 servings	11 (52.4)	3 (20.0)
None	1 (4.8)	2 (13.3)
Missing	5 (23.8)	1 (6.7)
Fresh fruit consumption (times/week)		
Daily	4 (19.0)	1 (6.7)
3-4 times per week	4 (19.0)	6 (40.0)
1-2 times per week	8 (38.1)	7 (46.7)
Never	1 (4.8)	1 (6.7)
Missing	4 (19.0)	0 (0)
Mean (SD)	3.17 (2.4)	2.57 (1.7)
Canned fruit consumption (times/week)		
1-2 times a week	8 (38.1)	9 (60.0)
Never	9 (42.9)	6 (40.0)
Missing	4 (19.0)	0 (0)
Mean (SD)	0.70 (0.8)	0.60 (0.7)
Frozen fruit consumption (times/week)		
1-2 times a week	6 (28.6)	7 (46.7)
Never	10 (47.6)	8 (53.3)
Missing	5 (23.8)	0 (0)
Mean (SD)	0.56 (0.8)	0.70 (0.8)
Types of fruits consumed		
Apples	14 (66.7)	11(73.3)
Bananas	9 (42.9)	7 (46.7)
Berries	12 (57.1)	13 (86.7)
Citrus fruits (oranges, lemons, etc.)	10 (47.6)	11 (73.3)
Other (grape, mango, pineapple)	1 (4.8)	2 (8.0)
Total vegetable servings (servings/day)		
3 – 4 servings	5 (23.8)	3 (20.0)
1 – 2 servings	10 (47.6)	12 (80.0)
Missing	6 (28.6)	0 (0)
Fresh vegetable consumption (times/week)		
Daily	5 (23.8)	1 (6.7)
3-4 times per week	6 (28.6)	8 (53.3)
1-2 times per week	5 (23.8)	1 (6.7)
Never	1 (4.8)	1 (6.7)
Missing	4 (19.0)	4 (26.7)
Mean (SD)	3.74 (2.4)	3.38 (1.6)

Canned vegetable consumption (times/week)	3 (14.3)	3 (20.0)
3-4 times a week	5 (23.8)	6 (40.0)
1-2 times a week	6 (28.6)	6 (40.0)
Never	7 (33.3)	0 (0)
Missing	1.18 (1.4)	1.8 (0.8)
Mean (SD)		
Frozen vegetable consumption (times/week)		
Daily	1 (4.8)	1 (6.7)
3-4 times a week	5 (23.8)	3 (20.0)
1-2 times a week	5 (23.8)	8 (53.3)
Never	4 (19.0)	3 (20.0)
Missing	6 (28.6)	0 (0)
Mean (SD)	4.13 (2.6)	4.73 (3.8)
Types of vegetables consumed		
Leafy greens (spinach, kale, lettuce)	15 (71.4)	14 (93.3)
Cruciferous vegetables (broccoli, cauliflower)	13 (61.9)	12 (80.0)
	16 (76.2)	13 (86.7)
Root vegetables (carrots, potatoes)	8 (38.1)	10 (66.7)
Bell peppers	1 (4.8)	0 (0)
Other (brussels, asparagus)		

Table S5 – Eating, Cooking and Food Purchase Behaviors of College Students Attending a Culinary Medicine Class at a Public University in Southern U.S [n=25; N=21 (baseline) and N=15 (endline)]

Characteristics	Baseline N (%)	Endline N (%)
Number of meals per day		
1	3 (14.3)	2 (13.3)
2	7 (33.3)	10 (66.7)
3	6 (28.6)	3 (20.0)
≥4	1 (4.8)	0 (0)
Missing	4 (19.0)	0 (0)
Mean (SD)	2.30 (0.8)	2.07 (0.6)
Number of snacks per day		
1	5 (23.8)	6 (40.0)
2	8 (38.1)	5 (33.5)
3	1 (4.8)	4 (26.7)
≥4	2 (9.5)	0 (0)
Missing	5 (23.8)	0 (0)
Mean (SD)	2.23 (1.3)	1.87 (0.8)
Meals skipped frequency		
Daily	8 (38.1)	8 (53.3)
Weekly	4 (19.0)	2 (13.3)
Bi-weekly	3 (14.3)	2 (13.3)
Rarely	1 (4.8)	3 (20)
Missing	5 (23.8)	13 (52)
Meal skipped most often		
Breakfast	8 (38.1)	8 (53.3)
Lunch	6 (28.6)	6 (40.0)
Dinner	2 (9.5)	0 (0)
Missing	5 (23.8)	1 (6.7)
Where meals are eaten (pick all that apply)		
Home	14 (66.7)	15 (100)
Off-campus restaurants	3 (14.3)	5(33.3)
Dining hall	2 (9.5)	7 (46.7)
On-campus retail locations	1 (4.8)	1 (6.7)
Café and Coffee shop	3 (14.3)	4 (26.7)
Cooking method (pick all that apply)		
Stove-top cooking	15 (71.4)	14 (93.3)
Micro-wave cooking	8 (38.1)	7 (46.7)
Micro-wave (heat and eat)	7 (33.3)	8 (53.3)
Bake	8 (38.1)	8 (53.3)
No cooking required (e.g. toss salads, sandwiches)	8 (38.1)	9 (60.0)
Air fryer	1 (4.8)	1 (6.7)

Receiving SNAP		
Yes	0 (0)	0 (0)
No	16 (76.2)	15 (100)
Missing	5 (23.8)	0 (0)
Location where food is purchased		
Grocery Store	15 (71.4)	15 (100.0)
Do not shop (Dining hall)	1 (4.8)	0 (0)
Missing	5 (23.8)	0 (0)
Frequency of receiving food via food giveaway		
Monthly	1 (4.8)	0 (0)
Rarely	2 (9.5)	3 (20.0)
Never	14 (66.7)	12 (80.0)
Missing	4 (19.0)	0 (0)
Food pantry visit frequency		
Bi-weekly	1 (4.8)	1 (6.7)
Monthly	1 (4.8)	1 (6.7)
Never	15 (71.4)	13 (86.7)
Missing	4 (19.0)	0 (0)
Received formal training in knife skills or Culinary Arts		
Yes	4 (19.0)	10 (66.7)
No	11 (52.4)	3 (20.0)
Missing	6 (28.6)	2 (13.3)
Use of knife (confidence rating)		
Very confident	7 (33.3)	6 (40.0)
High confidence	4 (19.0)	4 (26.7)
Moderate confidence	4 (19.0)	3 (20.0)
Little Confidence	1 (4.8)	1 (6.7)
Missing	5 (23.8)	1 (6.7)

Table S6 - Food Security Status and Mental Health Indicators of College Students Attending a Culinary Medicine Class at a Public University in Southern U.S (n=11)

Outcomes	Baseline N (%)	Endline N (%)	P-value
Food security			
Low food security (score 3-5)	9 (81.8)	8 (72.7)	
Very low food security (score 6-10)	2 (18.2)	3 (27.3)	
Mean (SD)	5.72 (1.7)	5.45 (0.9)	§0.50
Perceived stress scale			
Low stress (score 0–13)	1 (9.1)	3 (27.3)	
Moderate stress (score 14–26)	8 (72.7)	6 (54.5)	
High stress (score 27–40)	2 (18.2)	2 (18.2)	
Mean (SD)	19.27 (7.7)	17.54 (7.5)	§0.42
Generalized anxiety disorder scale			
Minimal Anxiety (score 0-4)	3 (27.3)	4 (36.4)	
Mild Anxiety (score 5-9)	5 (45.5)	5 (45.5)	
Moderate Anxiety score (10-14)	2 (18.2)	1 (9.1)	
Severe Anxiety (score >=15)	1 (9.1)	1 (9.1)	
Mean (SD)	7.73 (5.4)	7.00 (5.4)	§0.58
Connor-Davidson resilience scale			
High resilience (score >25.5)	8 (72.7)	8 (72.7)	
Low resilience (score <= 25.5)	3 (27.3)	3 (27.3)	
Mean (SD)	32.72 (9.7)	33.30 (9.9)	§0.22
Center for epidemiologic studies depression scale			
Low Depression risk (score <16)	8 (72.7)	9 (81.8)	
At Risk for Depression (score >=16)	3 (27.3)	2 (18.2)	
Mean (SD)	17.67 (14.9)	17.00 (14.3)	§0.27
Coping orientation to problems experienced inventory [Mean (SD)]			
Problem Focused Coping (score 1-4)	2.58 (0.8)	2.54 (0.7)	§0.34
Emotion Focused Coping (score 1-4)	2.23 (0.7)	2.31 (0.7)	§0.65
Avoidant Coping (score 1-4)	1.75 (0.6)	1.74 (0.7)	§0.88

§ P-value for Wilcoxon signed rank test

Table S7 - Fruit and Vegetable Consumption among College Students Attending a Culinary Medicine Class at a Public University in Southern U.S. (n=11)

**Outcomes**	**Baseline N (%)**	**Endline N (%)**	
Total fruit servings (serving/day)			
3-4 servings	3 (27.3)	2 (18.2)	
1-2 servings	6 (54.5)	7 (63.6)	
None	0 (0)	2 (18.2)	
Missing	2 (18.2)	0 (0)	†0.98
Fresh fruit consumption (times/week)			
Daily	2 (18.2)	1 (9.1)	
3-4 times a week	3 (27.3)	5 (45.5)	
1-2 times a week	5 (45.5)	4 (36.4)	
Never	0 (0)	1 (9.1)	
Missing	1 (9.1)	0 (0)	
Mean (SD)	3.2 (2.2)	2.77 (1.9)	§0.73
Canned fruit consumption (times/week)			
1-2 times a week	5 (45.5)	7 (64)	
Never	5 (45.5)	4 (36)	
Missing	1 (9.1)	0 (0)	
Mean (SD)	0.75 (0.8)	0.95(0.8)	§0.59
Frozen fruit consumption (times/week)			
1-2 times a week	4 (36.4)	5 (45.5)	
Never	5 (45.5)	6 (54.5)	
Missing	2 (18.2)	0 (0)	
Mean (SD)	0.67 (0.8)	0.68 (0.8)	§0.49
Types of fruits consumed			
Apples	9 (81.8)	8 (72.7)	
Bananas	6 (54.5)	5 (45.5)	
Berries	8 (72.7)	10 (90.9)	
Citrus fruits (oranges, lemons, etc.)	6 (54.5)	8 (72.7)	
Other (grape, mango, pineapple)	1 (9.1)	2 (18)	
Total vegetable servings (servings/day)			
3-4 servings	4 (36.4)	8 (72.7)	
1-2 servings	5 (45.5)	3 (27.3)	
Missing	2 (18.2)	0 (0)	†*0.03
Fresh vegetable consumption (times/week)			
Daily	3 (27.3)	1 (9.1)	

3-4 times a week	4 (36.4)	4 (36.4)	
1-2 times a week	3 (27.3)	5 (45.5)	
Never	0 (0)	1 (9.1)	
Missing	1 (9.1)	0 (0)	
Mean (SD)	3.95 (2.2)	2.59 (1.9)	§*0.04
Canned vegetable consumption			
(times/week)	3 (27.3)	2 (18.2)	
3-4 times a week	3 (27.3)	5 (45.5)	
1-2 times a week	3 (27.3)	4 (36.4)	
Never	2 (18.2)	0 (0)	
Missing	1.67 (1.5)	1.30 (1.3)	§0.08
Mean (SD)			
Frozen vegetable consumption (times/week)			
Daily	1 (9.1)	1 (9.1)	
3-4 times a week	4 (36.4)	2 (18.2)	
1-2 times a week	2 (18.2)	6 (54.5)	
Never	3 (27.3)	2 (18.2)	
Missing	1 (9.1)	0 (0)	
Mean (SD)	4.15 (4.7)	4.72 (5.1)	§0.47
Types of vegetables consumed			
Leafy greens (spinach, kale, lettuce)	9 (81.8)	9 (81.8)	
Cruciferous vegetables (broccoli, cauli-	9 (81.8)	9 (81.8)	
flower)	9 (81.8)	9 (81.8)	
Root vegetables (carrots, potatoes)	4 (36.4)	9 (81.8)	
Bell peppers	1 (9.1)	0 (0)	
Other (brussels, asparagus)			

*Significant at $p < .05$.

§ P-value for Wilcoxon signed rank test

† P-value for Fisher's exact test

No p-value where participants could select more than one option

Table S8 – Eating, Cooking and Food Purchase Behaviors of College Students Attending a Culinary Medicine Class at a Public University in Southern U.S (n=11)

Characteristics	Baseline N (%)	Endline N (%)	
Number of meals per day			
1	2 (18.2)	1 (9.1)	
2	4 (36.4)	7 (63.6)	
3	4 (36.4)	3 (27.3)	
Missing	1 (9.1)	0 (0)	
Mean (SD)	2.2 (0.8)	2.18 (0.6)	§0.91
Number of snacks per day			
1	4 (46.4)	5 (45.5)	
2	4 (36.4)	3 (27.3)	
3	1 (9.1)	3 (27.3)	
≥4	1 (9.1)	0 (0)	
Missing	1 (9.1)	0 (0)	
Mean (SD)	1.9 (0.9)	1.82 (0.8)	§0.34
Meals skipped frequency			
Daily	5 (45.5)	4 (36.4)	
Weekly	2 (27.3)	2 (18.2)	
Bi-weekly	1 (9.6)	3 (27.3)	
Rarely	2 (18.2)	2 (18.2)	
Missing	1 (9.1)	0 (0.0)	†0.10
Meal skipped most often			
Breakfast	5 (45.5)	6 (54.5)	
Lunch	4 (36.4)	5 (45.5)	
Dinner	1 (9.1)	0 (0)	
Missing	1 (9.1)	0 (0)	†0.06
Where meals are eaten (pick all that apply)			
Home	8 (72.7)	10 (90.9)	
Off-campus restaurants	1 (9.1)	2 (18.2)	
Dining hall	1 (9.1)	1 (9.1)	
On-campus retail locations	1 (9.1)	1 (9.1)	
Cooking method (pick all that apply)			
Stove-top cooking	9 (81.9)	11(100.0)	
Micro-wave cooking	6 (54.5)	4 (36.4)	
Micro-wave (heat and eat)	5 (45.5)	6 (54.5)	

Bake	4 (36.4)	8 (72.7)	
No cooking required (e.g. toss salads, sandwiches)	4 (36.4)	6 (54.5)	
Air fryer	1 (9.1)	1 (9.1)	
Receive SNAP			
Yes	0 (0)	0 (0)	
No	10 (90.9)	11 (100)	
Missing	1 (9.1)	0 (0)	
Location where food is purchased			
Grocery Store	10 (90.9)	11 (100.0)	
Missing	1 (9.1)	0 (0)	
Frequency of receiving food from a food giveaway			
Rarely	1 (9.1)	1 (9.1)	
Never	9 (81.8)	10 (90.9)	
Missing	1 (9.1)	0 (0)	†0.10
Food pantry visit frequency			
Bi-weekly	1 (9.1)	1 (9.1)	
Monthly	0 (0)	1 (9.1)	
Rarely	2 (18.2)	0 (0)	
Never	7 (63.6)	9 (81.8)	
Missing	2 (18.2)	0 (0)	†0.07
Received formal training in knife skills or culinary arts			
Yes	3 (27.3)	8 (72.7)	
No	6 (54.5)	2 (18.2)	
Missing	1 (9.1)	1 (9.1)	†0.58
Use of knife confidence rating			
High confidence	2 (18.2)	4 (36.4)	
Very confident	5 (45.5)	5 (45.5)	
Moderate confidence	3 (27.3)	2 (18.2)	
Missing	1 (9.1)	0 (0)	†0.60

§ P-value for Wilcoxon signed rank test

† P-value for Fisher's exact test

No p-value where participants could select more than one option

Table S9: Exploratory Spearman Correlations Between Endline Food Security and Selected Outcomes

Variables Compared	Spearman's ϱ	p-value
Endline Food Security & GPA	0.11	0.75
Endline Food Security & Depression	0.30	0.48
Endline Food Security & Perceived stress	0.39	0.26
Endline Food Security & Knife Skills Confidence	0.30	0.38
Endline Food Security & Number of meals	0.26	0.45
Endline Food Security & Number of snacks	0.32	0.67

Disclaimer/Publisher's Note: The statements, opinions and data contained in all publications are solely those of the individual author(s) and contributor(s) and not of MDPI and/or the editor(s). MDPI and/or the editor(s) disclaim responsibility for any injury to people or property resulting from any ideas, methods, instructions or products referred to in the content.
